# Expanding access to sodium-glucose cotransporter 2 inhibitors (SGLT2i) in the Ministry of Health Malaysia – a multiple HTA approach

**DOI:** 10.1017/S0266462324000643

**Published:** 2024-12-05

**Authors:** Coleen Siew Bee Choo, Yee Vern Yong, Haarathi Chandriah, Nur Sufiza Ahmad

**Affiliations:** 1 Pharmaceutical Services Programme, Ministry of Health Malaysia Petaling Jaya, Malaysia; 2 Dato’ Keramat Primary Healthcare Clinic, Ministry of Health Malaysia Kuala Lumpur, Malaysia

**Keywords:** decision-making, SGLT2i, technology assessment

## Abstract

**Objectives:**

Ministry of Health (MOH) Malaysia stakeholders seek primary care access to sodium-glucose cotransporter 2 inhibitor (SGLT2i). Addressing this required a complex decision, selecting among three SGLT2i for two different indications and two practice settings. The options include expanding the existing SGLT2i (empagliflozin) in the MOH Medicines Formulary to primary care and/or having dapagliflozin and/or luseogliflozin as alternatives. This study aimed to conduct a multiple health technology assessment (HTA) to determine the SGLT2i of choice for the MOH setting.

**Methods:**

The clinical benefits of SGLT2i were assessed through a systematic literature review and affordability was assessed through the development of three budget impact analysis models simulating seventy scenarios. Each model varied by prescribing indications, restrictions, and SGLT2i involved (M1: glycemic control, HbA1c between 6.5 percent and 10 percent, empagliflozin–dapagliflozin–luseogliflozin; M2: cardiovascular benefits, HbA1c less than 10 percent, empagliflozin-dapagliflozin; M3: a composite of M1 and M2). The outcome of the HTA was presented to the MOH decision-makers.

**Results:**

Although there was no significant difference in glycemic control between the SGLT2i, differences exist in cardiovascular benefits conferred. Despite having scenarios with lower net budget impact (NBI) in the M1, M2, and M3 models, decision-makers decided to expand empagliflozin use to primary care setting and add dapagliflozin for hospital-only setting for both indications [NBI of $4.38 mil] due to empagliflozin’s advantage in reducing risk for cardiovascular death and prior experience of its use in MOH.

**Conclusions:**

The multiple HTA approach guided the complex decision-making process by providing a holistic understanding of the decision’s impact.

## Introduction

Diabetes is a major public health concern causing considerable socioeconomic burden worldwide. In Malaysia, the prevalence of diabetes is increasing with eighteen percent of adults found to have elevated blood sugar levels in 2019 (1). The majority of diagnosed cases are type 2 diabetes mellitus (T2DM) (>ninety percent) (2) and were receiving treatment at public healthcare facilities managed by the Ministry of Health (MOH) (eighty-three percent) (1). This is of concern as diabetes is a major risk factor for cardiovascular and microvascular complications.

Recent evidence on cardiovascular benefits conferred by sodium-glucose cotransporter 2 inhibitors (SGLT2i) which extend beyond glycemic efficacy has caused a paradigm shift in diabetes management (3–6). Although glycated hemoglobin A1c (HbA1c) remains an important target, emphasis is now given to selecting pharmacologic agents which can also address patients’ cardiovascular and renal risks (7–9). Hence, SGLT2i is now the recommended second-line treatment option after metformin for T2DM patients with underlying cardiovascular disorders and diabetic kidney disease (7–9).

In Malaysia, the MOH Medicines Formulary (MOHMF) (i.e., list of medicines approved for use in MOH facilities) has only one SGLT2i listed which is empagliflozin. Empagliflozin is approved for glycemic control and risk reduction of cardiovascular death in T2DM patients with established cardiovascular disease. However, its use is limited to patients managed in the hospital setting.

Following increasing evidence on the cardiovascular benefits of SGLT2i, stakeholders have requested to widen access to SGLT2i in the primary care setting. This can be achieved by either (1) expanding access to empagliflozin to the primary care setting (for either one or both currently MOHMF listed indications), and/or (2) having either dapagliflozin and/or luseogliflozin as an alternative, new SGLT2i for glycemic control, and/or (3) have dapagliflozin as an alternative for cardiovascular risk reduction. In the latter two scenarios, the use of dapagliflozin and/or luseogliflozin can be either restricted to the hospital setting or expanded to the primary care thus, creating multiple decision scenarios for consideration by the decision-makers.

In MOH Malaysia, the MOH Medicines Formulary Panel (MOHMFP) decides whether to list a new drug or expand the use of an existing drug. This decision is made through deliberation on the new drug’s comparative safety, efficacy, effectiveness, and budget implication relative to existing alternatives in the MOHMF. Additionally, the Panel considers factors such as unmet needs, patient-reported outcomes, and organizational issues.

The standard formulary listing approach assesses a single drug for a single indication, though occasionally multiple drugs sharing the same indication may be assessed together. In the case of SGLT2i, decision-makers need to select among three SGLT2i for two indications and two practice settings. This complexity requires an alternative approach to the standard health technology assessment (HTA) to determine the most beneficial option for the MOH setting. This study aimed to conduct a multiple HTA to determine SGLT2i of choice for MOH setting.

## Methods

The HTA of SGLT2i comprises two key components; a systematic literature review on published clinical evidence and budget impact analysis (BIA) to assess the affordability of expanding access to SGLT2i in MOH.

### Systematic Literature Review: Clinical Evidence

#### Search Strategy

As luseogliflozin is indicated only for glycemic control, two separate reviews were conducted to assess the comparative efficacy and safety between (1) empagliflozin, dapagliflozin, and luseogliflozin for glycemic control, and (2) empagliflozin and dapagliflozin for cardiovascular risk reduction.

Systematic literature searches were conducted in MEDLINE (PubMed) and Cochrane Library for each of the indications on 12 January 2022. The search was conducted using a combination of Medical Subject Heading (MeSH) and keywords with a limit to articles published in the English language between 2012 and 12 January 2022 [Supplementary Materials 1]. The search was supplemented with articles submitted by the respective product registration holders, articles found by focused Internet search and manual screening of the reference lists of all identified studies.

#### Selection Criteria

Titles and abstracts were screened by two independent reviewers (CCSB, YYV) before screening the full-text articles for final inclusion into the review. Any disagreement was resolved by consensus, and if necessary, in consultation with a third reviewer (HC). Studies fulfilling the inclusion and exclusion criteria outlined in Supplementary Materials 2 were considered eligible.

#### Data Extraction and Quality Appraisal

Two reviewers (CCSB, YYV) appraised and extracted data from the selected studies using a standardized format. The data extracted include author, year of publication, study design, participants’ characteristics, sample size, intervention, comparator, study period or length of follow-up and outcomes. The methodological quality of the studies was assessed using relevant appraisal tools [e.g., Critical Appraisal Skills Programme (CASP) randomized controlled trial (RCT) checklist, and A MeaSurement Tool to Assess systematic Review (AMSTAR) 2 checklist for meta-analysis (MA)]. Discrepancies during data extraction and quality appraisal were resolved between the reviewers through discussion, and if necessary, in consultation with a third reviewer (HC).

### BIA

The budget implications of listing new SGLT2i into the MOHMF and/or expanding the use of SGLT2i to primary care setting for the proposed indications were assessed using BIA. To aid decision-making, three separate BIA models were developed using Microsoft Excel in accordance with the report of the ISPOR Task Force on Good Research Practices — BIA (10). The M1 model estimates the budget implication for using empagliflozin, dapagliflozin and/or luseogliflozin in various combinations for glycemic control in adult T2DM patients with HbA1c levels between 6.5 percent and 10 percent whereas the M2 model estimates the budget implication for using empagliflozin and/or dapagliflozin for cardiovascular risk reduction in adult T2DM patients with HbA1c level below 10 percent and have established cardiovascular disease. In each of the BIA, the use of individual SGLT2i may be restricted to hospital setting or expanded to primary care and were estimated over a time horizon of 5 years. In view of the significant overlap in the patient population between the two indications, a third BIA (M3) which simulates scenarios for adopting at least a change each in the availability of SGLT2i for glycemic control and cardiovascular risk reduction, respectively was also modeled to better quantify the budget implication resulting from the decisions made. In total, seventy different SGLT2i uptake scenarios which also reflected the volume-based pricing for dapagliflozin were modelled. The outcome of the BIA was expressed as net budget impact (NBI) or incremental budget, reflecting a difference in total budget between current and future scenarios. All costs were expressed in United States Dollars ($) based on an exchange rate of $1 equivalent to Malaysian Ringgit 0.227 in July 2022.

### Deliberation of Evidence

The evidence on the comparative efficacy and safety between the SGLT2i in glycemic control and cardiovascular risk reduction as well as the budget implication from the adoption of new and/or expanding access to SGLT2i was presented to the MOHMFP to aid decision-making.

## Results

### Search Results and Quality of Studies

**Figure 1.**
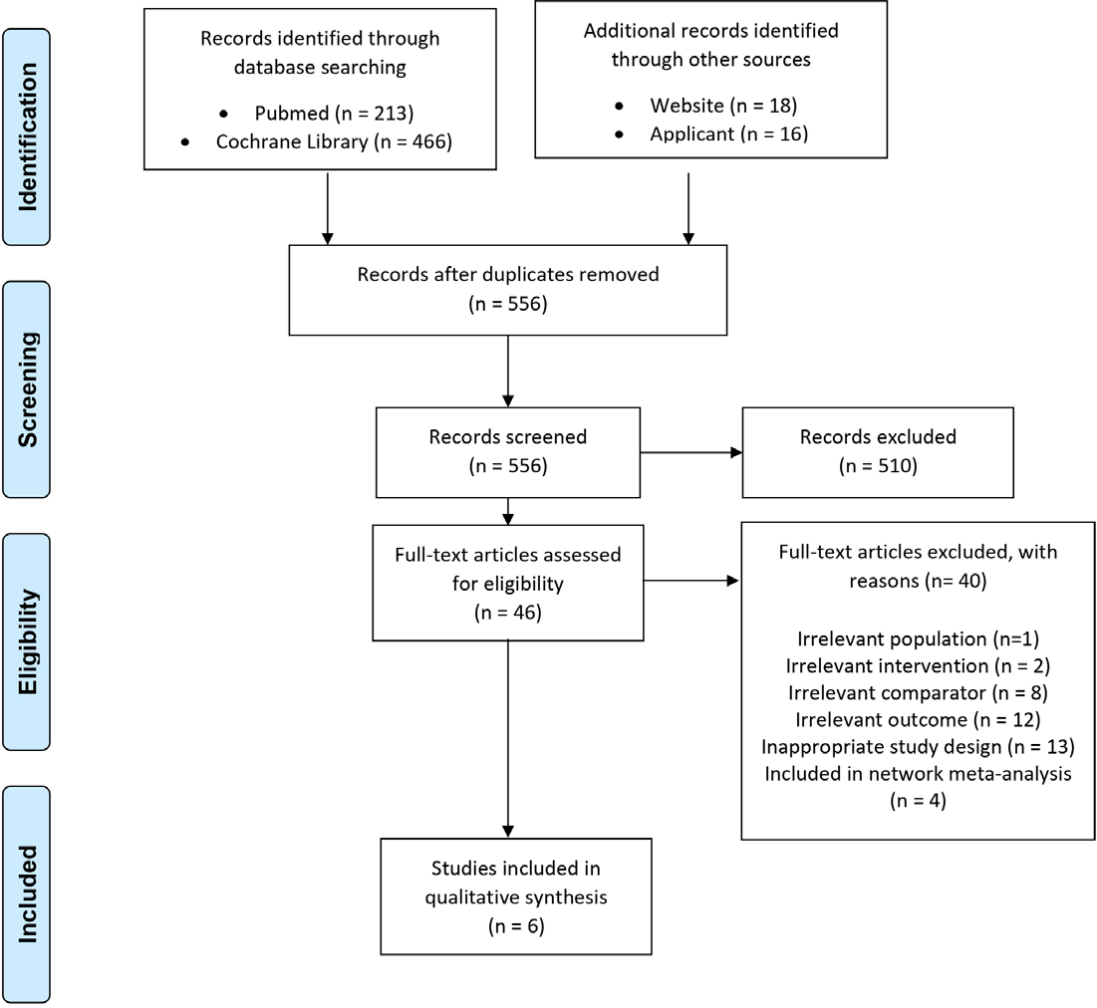
PRISMA flow diagram of study selection for glycemic control.

The search and selection of studies for the use of SGLT2i in glycemic control is outlined in Figure 1 and cardiovascular risk reduction is outlined in Supplementary Materials 3: Figure S1. The review included six studies assessing the comparative safety and efficacy of SGLT2i for glycemic control and eighteen studies for cardiovascular risk reduction, all with low to moderate risk of bias. The characteristics of the included studies are summarized in Supplementary Materials 3: Table S1.

### Efficacy of SGLT2i for Glycemic Control

Three network meta-analyses (NMAs) were identified providing evidence of the comparative efficacy of SGLT2i in glycemic control when given as a combination therapy (11–13). A comprehensive NMA reported on the efficacy of various glucose-lowering drugs (GLDs) when added to metformin in the treatment of T2DM (sixty-two RCTs; 32,185 participants). In this study, dapagliflozin was found to be inferior to empagliflozin in reducing HbA1c level [weighted mean difference (WMD) 0.21 percent (95 percent Confidence Interval (CI): 0.02 to 0.39 percent) for comparison of dapagliflozin to empagliflozin] (11). However, this difference was not clinically significant [clinical superiority is defined as HbA1c improvement by at least 0.3 percent] (14).

A contrasting NMA included only RCTs involving SGLT2i (three RCTs; 2,455 participants). This study found no significant difference between dapagliflozin 10 mg daily and empagliflozin 25 mg daily in HbA1c control when given in combination with metformin [WMD 0.10 percent (95 percent Credible Interval (CrI): −0.14 percent to 0.34 percent)] (12). In the same study, the proportion of patients achieving HbA1c less than seven percent was also comparable between dapagliflozin 10 mg daily and empagliflozin 25 mg daily [risk ratio (RR) 0.81 (95 percent CrI: 0.60 to 1.06)].

Likewise, in another NMA which pooled evidence from RCTs involving the use of SGLT2i as monotherapy and add-on combination therapy, dapagliflozin 10 mg daily was found to have comparable efficacy to empagliflozin 25 mg daily in both HbA1c (thirty-eight RCTs; 23,997 participants) and fasting plasma glucose control (thirty-seven RCTs; 19,491 participants) (13). The difference in outcomes between dapagliflozin and empagliflozin from these NMAs is likely attributed to the difference in inclusion criteria for RCTs employed in these analyses. However, taken together, there are likely no clinically significant differences between dapagliflozin and empagliflozin in terms of glycemic control [refer Table 1].

**Table 1.** Summary of comparative efficacy between SGLT2i in glycemic control & weight loss

Outcomes measured	EMPA vs. DAPA	EMPA vs. LUSE		DAPA vs. LUSE
**HbA1c reduction, WMD(%)^11^** ^**–**^ **^13^** ^**;**^ **^15^**	↔ No ***clinically significant*** * difference *Clinically significant difference: Difference of at least 0.3% at the lower bound of 95% CI interval	↔ Likely no clinically significant difference		
		**Placebo-corrected changes from baseline**		**Change from baseline (Uncontrolled trial)**
		**DAPA + MTF**	**EMPA + MTF**	**LUSE + MTF**
		−0.48% to −0.54%	−0.64% to −0.69%	−0.61%
**Impact on weight, WMD(kg)^11^** ^**–**^ **^13^** ^**;**^ **^15^**	↔ No significant difference	↔ Likely no significant difference		
		**Placebo-corrected changes from baseline**		**Change from baseline (Uncontrolled trial)**
		**DAPA + MTF**	**EMPA + MTF**	**LUSE + MTF**
		−2.17 kg	−2.08 kg	−2.86 kg

Abbreviations: DAPA – dapagliflozin; EMPA – empagliflozin; LUSE – luseogliflozin; MTF – metformin; WMD – weighted mean difference.

There were no direct head-to-head comparisons or indirect comparisons between luseogliflozin with either dapagliflozin or empagliflozin as an add-on therapy to other GLDs. Nevertheless, evidence from an open-label trial showed that the HbA1c reduction from baseline for luseogliflozin 2.5 mg daily in combination with metformin after 52 weeks was −0.61 percent (95 percent CI: not reported) (15). This is comparable to the HbA1c reduction seen with dapagliflozin 10 mg daily (mean difference with placebo: −0.48 percent to −0.54 percent) and empagliflozin 25 mg daily (mean difference with placebo: −0.64 percent to −0.69 percent) when given in combination with metformin [refer Table 1] (11;12). Thus, there is unlikely any difference in glycemic control between luseogliflozin, dapagliflozin, and empagliflozin.

In comparison to placebo, both dapagliflozin and empagliflozin resulted in statistically significant weight loss when given in combination with metformin (WMD for dapagliflozin: −2.17 kg; empagliflozin: −2.08 kg) (11). However, evidence from NMAs showed that there is no significant difference in weight loss between dapagliflozin and empagliflozin when given as an add-on combination with metformin (11;12) [refer Table 1].

There was no comparative evidence between luseogliflozin and other SGLT2i for weight loss. In an open-label clinical trial, the weight loss after 52 weeks of treatment using luseogliflozin plus metformin was reported to be −2.86 kg (15).

### Efficacy of SGLT2i for Cardiovascular Risk Reduction

Evidence supporting the cardiovascular benefits of empagliflozin and dapagliflozin among patients with T2DM comes from the landmark – EMPA-REG OUTCOME and DECLARE-TIMI 58 trials (3;4). The EMPA-REG OUTCOME trial tested 7,020 participants; 4,687 were randomized to empagliflozin, with a median follow-up of 3.1 years. In this trial, empagliflozin when added to the standard of care (SoC) was superior to SoC alone in reducing 3-point major adverse cardiovascular events (MACE) among T2DM patients with established atherosclerotic cardiovascular disease (ASCVD). MACE was defined as a composite of death from cardiovascular causes, nonfatal myocardial infarction or nonfatal stroke. There was a fourteen percent reduction in the risk for 3-point MACE among patients treated with empagliflozin [Hazard Ratio (HR) 0.86 (95 percent CI: 0.74 to 0.99); *p* = 0.04 for superiority] with effect largely driven by a significant reduction in cardiovascular death [HR 0.62 (95 percent CI: 0.49 to 0.77); *p* <0.001] (3).

Similarly, there was also a significant reduction in the risk of hospitalization due to heart failure (HHF) by thirty-five percent [HR 0.65 (95 percent CI: 0.50 to 0.85); *p* = 0.002] and all-cause mortality by thirty-two percent [HR 0.68 (95 percent CI: 0.57 to 0.82); *p* < 0.001] [Refer Table 2]. The cardiovascular benefits of empagliflozin were also confirmed in MAs (16–18) and maintained among Asian patients (19), patients with or without peripheral artery disease (PAD) (20), prior history of coronary artery bypass grafting (21), prevalent kidney disease (22), heart failure (23), risk for heart failure based on 9-variable Health ABC Heart Failure Risk Score (24) and Thrombolysis in Myocardial Infarction Risk Score for Heart Failure in Diabetes categories (TRS-HFDM) (25), history of myocardial infarction or stroke (26), and cardiovascular risk factor control at baseline (27).

**Table 2.** Summary of comparative efficacy between SGLT2i in cardiovascular risk reduction

Cardiovascular outcome trials	Relative risk reduction (%) vs. SoC			
	3-point MACE*	Cardiovascular death	All-cause mortality	Hospitalization due HF
**EMPA-REG OUTCOME trial** EMPAgliflozin 10 mg, 25 mg + SoC vs. SoC N = 7,020 T2DM patients **100% with ASCVD** 10.1% with HF	**↓** 14%	**↓** 38%	**↓** 32%	↓ 35%
**DECLARE-TIMI 58 trial** DAPAgliflozin 10 mg + SoC vs. SoC N = 17,160 T2DM patients **40.6% with ASCVD** 10% with HF	**Overall Pop’n:** No significant difference **Subgp with ASCVD:** No significant difference	**Overall Pop’n:** No significant difference **Subgp with ASCVD:** No significant difference	**Overall Pop’n:** No significant difference **Subgp with ASCVD:** No significant difference	**Overall Pop’n:** **↓** 27% **Subgp with ASCVD:** **↓** 22%

Abbreviations: *3-point MACE, major adverse cardiovascular events (composite of death from cardiovascular causes, nonfatal MI or nonfatal stroke); ASCVD, atherosclerotic cardiovascular disease; HF, heart failure; Pop’n, population; SoC, standard of care; Subgp, subgroup; T2DM, type 2 diabetes mellitus.

The DECLARE-TIMI 58 trial tested 17,160 participants; 8,582 randomized to dapagliflozin, with a median follow-up of 4.2 years. This trial tested the addition of dapagliflozin to SoC and unlike empagliflozin, found dapagliflozin did not reduce the rate of 3-point MACE [HR 0.90 (95 percent CI: 0.79 to 1.02)], cardiovascular death [HR 0.94 (95 percent CI: 0.76 to 1.18)], or all-cause mortality [HR 0.92 (95 percent CI: 0.79 to 1.08)] among T2DM patients with ASCVD (4). However, in this patient population, dapagliflozin significantly lowers the risk of HHF by twenty-two percent [HR 0.78 (95 percent CI: 0.63 to 0.97)] [Refer Table 2]. Although the cardiovascular outcomes for dapagliflozin were found to be consistent in a subgroup of patients with or without myocardial infarction (28) and PAD (29), patients with heart failure with reduced ejection fraction (HFrEF) were found to have a lower rate of cardiovascular death and all-cause mortality than those without HFrEF (*p*-interaction between subgroups < 0.05) (30). In contrast to the DECLARE-TIMI 58 trial, evidence from early MAs showed that there was no significant difference in the rate of heart failure or HHF with dapagliflozin (17;31). However, in a recent NMA (twenty-four trials including ten cardiovascular outcome trials (CVOTs) and renal outcome trials; 62,044 participants), dapagliflozin significantly reduced the rate of HHF among patients with established cardiovascular disease by twenty-one percent [rate ratio 0.79 (95 percent CrI: 0.64 to 0.97)] (18).

Evidence from NMA showed no significant difference between dapagliflozin and empagliflozin in the risk for 3-point MACE, cardiovascular death, all-cause mortality and heart failure or HHF (18).

### Safety

Evidence from NMAs showed no significant difference between dapagliflozin and empagliflozin in the risk for hypoglycemia, genital tract infections (11;13), and bone fractures (32;33) [Supplementary Materials 3: Table S2]. In an NMA assessing the efficacy of various GLDs when added to metformin, there appears to be no significant difference between dapagliflozin and empagliflozin in the rate of urinary tract infections (UTIs) [RR 1.50 (95 percent CI: 0.89 to 2.50)] (11). However, a separate NMA focusing only on RCTs of SGLT2i (both as monotherapy and in combination with other GLDs), showed the risk for UTIs is significantly higher for dapagliflozin 10 mg daily than empagliflozin 25 mg daily [odds ratio (OR) 1.39 (95 percent CI: 1.07 to 1.81)] (13). The comparative safety between the three SGLT2i in the risk for euglycemic ketoacidosis and amputation remained unknown due to the absence of published evidence. Despite the lack of published comparative safety evidence of luseogliflozin with the other SGLT2i, no clinically significant difference in safety profiles is anticipated between the three SGLT2i.

### BIA

**Figure 2.**
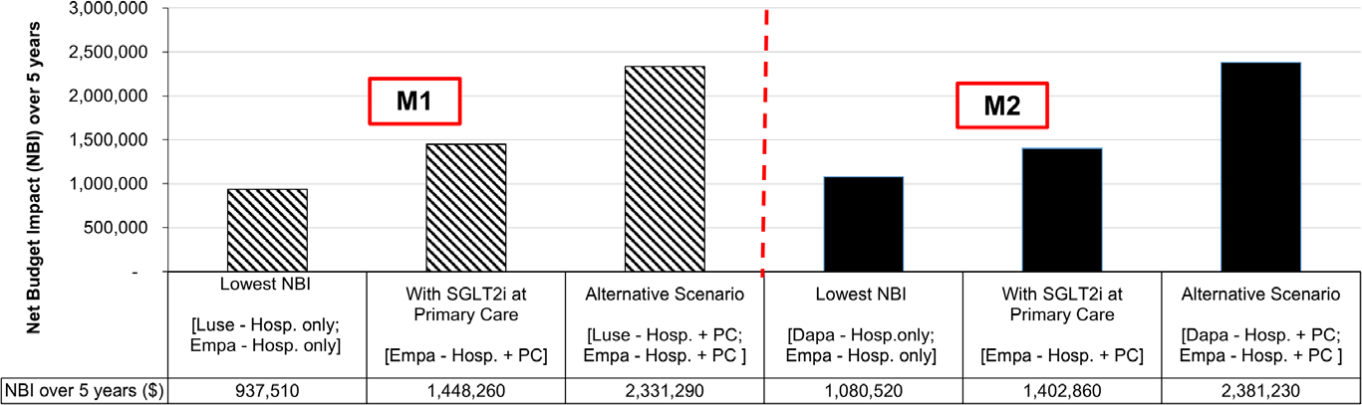
Budget impact analysis for glycemic control (M1) & cardiovascular risk reduction (M2). Abbreviations: Dapa, dapagliflozin; Empa, empagliflozin; Hosp., hospital setting; Luse, luseogliflozin; NBI, net budget impact; PC, primary care setting; SGLT2i, sodium glucose cotransporter 2 inhibitors.

Seventy scenarios were modeled but only selected scenarios of interest were presented to the MOHMFP. Alternative scenarios were presented upon request. Figure 2 shows three budget impact scenarios simulating the listing of new SGLT2i and/or expanding the use of SGLT2i to primary care setting for glycemic control (M1) and cardiovascular risk reduction (M2), respectively. In the M1 BIA, the scenario with the lowest NBI is to list luseogliflozin for a hospital setting only while maintaining the use of empagliflozin in a hospital setting (NBI: $0.94 mil). The scenario that addresses the request to expand the use of SGLT2i to primary care with the lowest NBI is to expand the use of empagliflozin to both hospital and primary care settings without listing any new SGLT2i (NBI: $1.45 mil). An alternative scenario which provides a selection of SGLT2i to prescribers in both practice settings (that is having at least two SGLT2i available) but with the lowest NBI will be listing luseogliflozin for hospital and primary care use while simultaneously expanding the use of empagliflozin to the same settings (NBI: $2.33 mil). Listing both dapagliflozin and luseogliflozin for hospital and primary care use while restricting the use of empagliflozin in the hospital setting incurred the highest NBI ($4.06 mil).

For cardiovascular risk reduction (M2), the NBI of the various budget impact scenarios ranges from $1.08 mil [dapagliflozin (new) and empagliflozin – both for a hospital setting only] to $2.48 mil [dapagliflozin (new) for hospital setting only and empagliflozin for hospital and primary care settings]. Among the scenarios which consider expanding the use of SGLT2i to primary care setting, expanding the use of empagliflozin alone without listing any new SGLT2i (NBI: $1.40 mil) incurred the lowest incremental budget whereas the alternative scenario of simultaneously listing both dapagliflozin and empagliflozin for use in hospital and primary care settings incurred a NBI of $ 2.38 mil.

**Figure 3.**
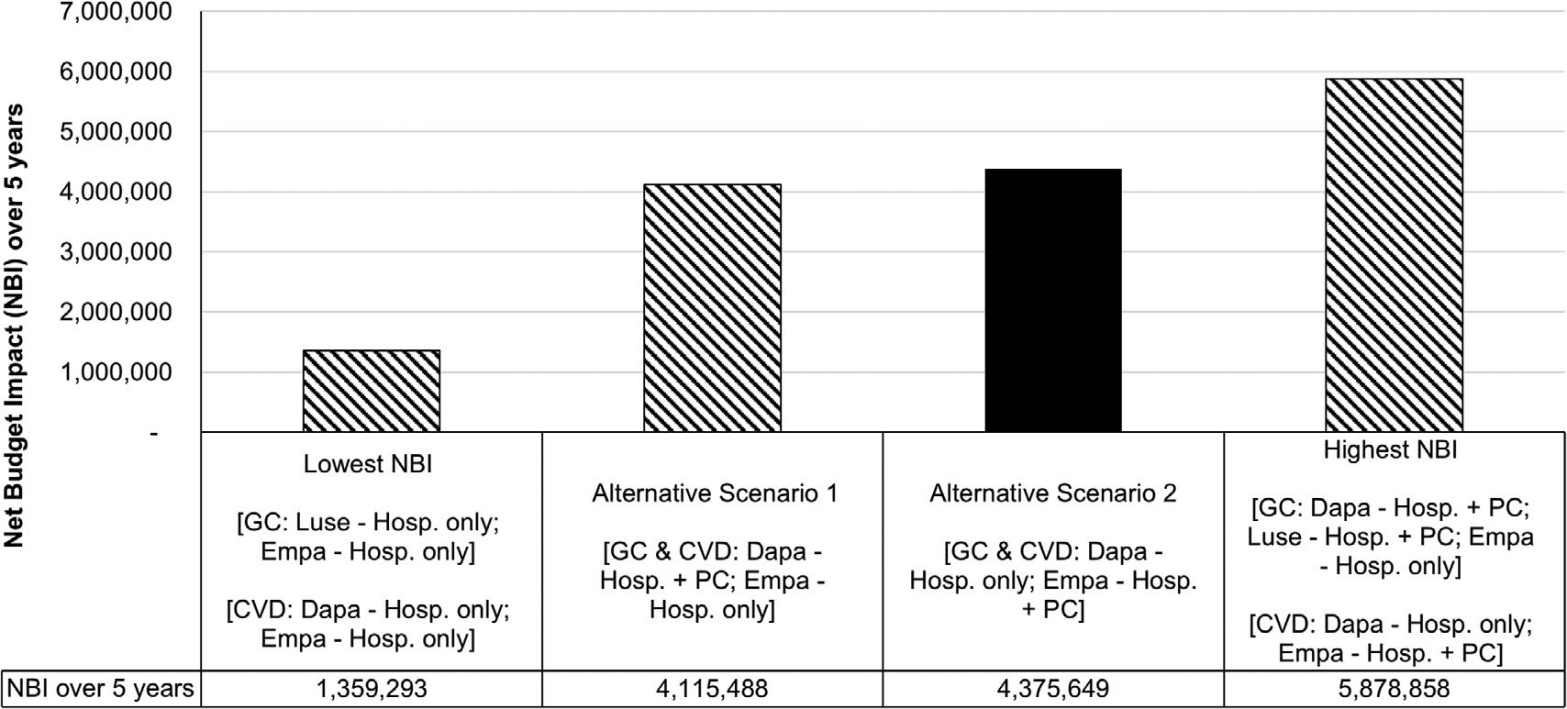
Budget impact analysis for a composite of glycemic control (M1) and cardiovascular risk reduction (M2) [M3 model]. Abbreviations: CVD, cardiovascular risk reduction; Dapa, dapagliflozin; Empa, empagliflozin; GC, glycemic control; Hosp., hospital setting; Luse, luseogliflozin; NBI, net budget impact; PC, primary care setting.

The M3 BIA simulated scenarios for adopting at least a change each in the availability of SGLT2i for glycemic control and cardiovascular risk reduction, respectively after accounting for overlap in patient population between the two indications. In the M3 model, the NBI of the seventy possible budget impact scenarios ranges from $1.36 mil to $5.88 mil [Figure 3]. Listing dapagliflozin for use in hospital and primary care settings for both glycemic control and cardiovascular risk reduction while restricting the use of empagliflozin in hospital setting will result in the incremental budget of $4.12 mil. Expanding the use of empagliflozin to the primary care (for both indications) while listing dapagliflozin as an alternative SGLT2i for both indications in the hospital setting only resulted in a higher NBI of $4.38 mil.

### Final Decision by MOHMFP

The outcomes of the review and BIA were presented to the MOHMFP. After appraisal and due deliberation on the evidence, the Committee recommended expanding the use of empagliflozin to the primary care setting for glycemic control and cardiovascular risk reduction. Dapagliflozin was also listed as an alternative SGLT2i for both indications but restricted for use in hospital settings only.

## Discussion

Various HTA agencies have recommended the use of SGLT2i in combination with other GLDs for T2DM (34–49). These recommendations are based on the proven efficacy of SGLT2i in improving glycemic control and weight reduction, their safety profile and cost-effectiveness compared to alternative GLDs. Early HTAs published between 2013 and 2017 focused primarily on glycemic control, with one specific HTA on empagliflozin for cardiovascular risk reduction (34–50). Most HTAs followed a standard single technology assessment approach, assessing and recommending individual SGLT2i for single indication (34–48,50). However, the Agency for Care Effectiveness (ACE) of Singapore conducted a combined appraisal of multiple SGLT2i as part of dual or triple therapy with other GLDs for glycemic control (49). Despite involving multiple SGLT2i, the appraisal was limited to one indication.

In Malaysia, diabetes is a growing public health concern, with sixty-eight percent of known diabetes patients receiving treatment in a primary care setting (1). Among them, six percent have ischaemic heart disease, two percent have cerebrovascular disease, and fourteen percent have nephropathy (2). Notably, the economic burden associated with the management of T2DM and its complications is significant in Malaysia (51;52). Expanding SGLT2i use in primary care would therefore offer significant cardiovascular and renal protection to these patients (3–6), potentially preventing complications and reducing the associated economic burden. The decision of which SGLT2i to select for treating these patients is complex because there are multiple SGLT2i licensed for different indications. Previous HTA appraisals focused on glycemic control evidence (34–49), requiring an update to reflect the new indications approved which is cardiovascular risk reduction. Furthermore, luseogliflozin was also not included in previous HTAs. Therefore, a multiple HTA approach was adopted to guide the complex decision by the MOHMFP involving three SGLT2i for two indications, across two practice settings. This approach differs from the conventional multiple technology appraisal (MTA) by the National Institute for Health and Care Excellence (NICE) of the United Kingdom, which appraises multiple technologies for a similar indication, or one technology for multiple indications (53). However, ACE Singapore recently published guidance on using SGLT2i for treating HFrEF and chronic kidney disease, also assessing multiple SGLT2i for multiple indications (54).

Our review of published evidence suggests that there is likely no clinically significant difference between dapagliflozin, empagliflozin and luseogliflozin in terms of glycemic control, weight reduction and overall safety profile (11–13). Hence for glycemic control, treatment cost or affordability of the respective SGLT2i would be the primary factor influencing the final formulary decision. However, there are differences in comparative effectiveness between the SGLT2i in terms of cardiovascular risk reduction. Although both dapagliflozin and empagliflozin have been shown to reduce the risk of HHF, only empagliflozin significantly reduced cardiovascular death when added to SoC in T2DM patients with established ASCVD (3;4).

Recognizing the macrovascular complications associated with T2DM, the MOHMFP acknowledged the importance of choosing an SGLT2i which can confer both glycemic control and cardiovascular benefits. Therefore, it was decided to expand SGLT2i use in primary care setting for both indications and include additional SGLT2i in the formulary to provide treatment options and encourage market price competition. As such, the M3 BIA which simulates scenarios of preference was used to guide the MOHMFP’s decision.

In the M3 BIA, listing luseogliflozin and dapagliflozin as alternative SGLT2i to empagliflozin for glycemic control and cardiovascular risk reduction, respectively in hospital setting resulted in the lowest NBI of $ 1.36 million [Figure 3]. Nevertheless, luseogliflozin was not selected as an alternative SGLT2i for formulary inclusion as it is not indicated for cardiovascular risk reduction. Moreover, this scenario also limits the availability of SGLT2i to the hospital setting. Therefore, listing dapagliflozin as a new SGLT2i for use in hospital and primary care settings for both indications while keeping empagliflozin for use in hospital setting [Figure 3: Alternative Scenario 1] was an attractive option. Despite a higher NBI, expanding empagliflozin use to primary care was preferred over dapagliflozin due to empagliflozin’s advantage in reducing risk for cardiovascular death and prior experience of its use in MOH [Figure 3: Alternative Scenario 2]. This shows that although cost was important, it was not the main driving factor for MOHMFP’s decision. Other factors such as indication coverage, comparative efficacy and prior experience in use were equally important in guiding MOHMFP’s decision.

If the conventional MTA approach of appraising multiple SGLT2i for single indication was adopted in our assessment, listing luseogliflozin for use in hospital and primary care settings alongside empagliflozin for glycemic control [Figure 2: M1 Alternative Scenario] and dapagliflozin for hospital and primary care settings alongside empagliflozin for cardiovascular risk reduction [Figure 2: M2 Alternative Scenario] could have been the possible decisions made by the MOHMFP to fulfil the requirement of having additional SGLT2i listed in the formulary and expanding use to the primary care setting. These decisions, however, would have posed challenges in prescribing SGLT2i as luseogliflozin cannot be prescribed for T2DM patients requiring cardiovascular protection. Our multiple HTA approach which simultaneously assessed two indications was able to highlight to the decision-makers that empagliflozin confers additional benefit of reducing risk of cardiovascular death over dapagliflozin. As such, despite being more expensive, expanding the use of empagliflozin in primary care setting for the treatment of T2DM was deemed the optimal decision.

Amidst rising healthcare costs, making evidence-informed formulary decisions is crucial. In this assessment, employing the multiple HTA approach which combined the appraisal of multiple SGLT2i for multiple indications in a single assessment has facilitated a holistic understanding of the available evidence by the decision-makers. This approach has aided them in making decisions which reflect the best use of limited healthcare resources.

## Limitation

There was no local cost-effectiveness study available to support the decision-making. Notably, cost-effectiveness evidence is not a mandatory requirement for the MOHMF decision-making. Local cost-effectiveness evidence would provide insights into the value of SGLT2i in the Malaysian context and can support price negotiation. As the use of SGLT2i is expanding to other indications, the conduct of local cost-effectiveness studies becomes increasingly important to inform future policy decisions. The BIA model only accounted for the cost of reduction in HHF, potentially underestimating the savings gained from reduction in cardiovascular death and diabetic nephropathy. As the inputs used in the BIA model such as cost and population estimates were specific to the Malaysian healthcare settings, the findings of this study may not be generalizable to other settings.

## Conclusion

Although single technology assessment is the standard approach engaged by various HTA agencies worldwide, our experience with SGLT2i indicates that a multiple HTA approach is more relevant in complex situations involving multiple drugs of the same class used in one or more indications. We anticipate that the multiple HTA approach will gain greater traction in the future as more drugs sharing similar indications become available.

## Supporting information

Choo et al. supplementary material 1 Choo et al. supplementary material

Choo et al. supplementary material 2 Choo et al. supplementary material

Choo et al. supplementary material 3 Choo et al. supplementary material

## Supplementary material

The supplementary material for this article can be found at http://doi.org/10.1017/S0266462324000643.
