# Supplementary material for: Expanding access to sodium-glucose cotransporter 2 inhibitors (SGLT2i) in the Ministry of Health Malaysia – a multiple HTA approach
Source: Int J Technol Assess Health Care. 2024 Dec 5;40(1):e69. doi: 10.1017/S0266462324000643 (PMC11703615; doi:10.1017/S0266462324000643)
Supplement: Choo et al. supplementary material 1 — Choo et al. supplementary material [file S0266462324000643sup001.docx]

**Supplementary Materials 1: Search strategies for MEDLINE (PubMed) and Cochrane Library databases**

1. **GLYCEMIC CONTROL**
   1. **Database searched: MEDLINE (PubMed) [Date searched: 12/1/2022]**

| **Search number** | **Search Details** | **Results** |
| --- | --- | --- |
| 8 | ((("diabetes mellitus, type 2"[MeSH Terms] AND "adult"[MeSH Terms] AND ("Sodium glucose transport protein 2 inhibitors"[Title/Abstract] OR "sglt2 inhibitor"[Title/Abstract] OR "dapagliflozin"[Title/Abstract] OR "empagliflozin"[Title/Abstract] OR "luseogliflozin"[Title/Abstract])) NOT ("glp"[Title/Abstract] OR "liraglutide"[Title/Abstract] OR "semaglutide"[Title/Abstract] OR "exenatide"[Title/Abstract] OR "albiglutide"[Title/Abstract] OR "dulaglutide"[Title/Abstract])) NOT ("cardiovascular system"[MeSH Terms] OR ("cardiovascular"[All Fields] AND "system"[All Fields]) OR "cardiovascular system"[All Fields] OR "cardiovascular"[All Fields] OR "cardiovasculars"[All Fields] OR ("heart failure"[MeSH Terms] OR ("heart"[All Fields] AND "failure"[All Fields]) OR "heart failure"[All Fields]) OR "kidney"[MeSH Terms])) AND ((y_10[Filter]) AND (meta-analysis[Filter] OR randomizedcontrolledtrial[Filter]) AND (humans[Filter]) AND (english[Filter])) | 213 |
| 7 | (("diabetes mellitus, type 2"[MeSH Terms] AND "adult"[MeSH Terms] AND ("Sodium glucose transport protein 2 inhibitors"[Title/Abstract] OR "sglt2 inhibitor"[Title/Abstract] OR "dapagliflozin"[Title/Abstract] OR "empagliflozin"[Title/Abstract] OR "luseogliflozin"[Title/Abstract])) NOT ("glp"[Title/Abstract] OR "liraglutide"[Title/Abstract] OR "semaglutide"[Title/Abstract] OR "exenatide"[Title/Abstract] OR "albiglutide"[Title/Abstract] OR "dulaglutide"[Title/Abstract])) NOT ("cardiovascular system"[MeSH Terms] OR ("cardiovascular"[All Fields] AND "system"[All Fields]) OR "cardiovascular system"[All Fields] OR "cardiovascular"[All Fields] OR "cardiovasculars"[All Fields] OR ("heart failure"[MeSH Terms] OR ("heart"[All Fields] AND "failure"[All Fields]) OR "heart failure"[All Fields]) OR "kidney"[MeSH Terms]) | 443 |
| 6 | "cardiovascular system"[MeSH Terms] OR ("cardiovascular"[All Fields] AND "system"[All Fields]) OR "cardiovascular system"[All Fields] OR "cardiovascular"[All Fields] OR "cardiovasculars"[All Fields] OR ("heart failure"[MeSH Terms] OR ("heart"[All Fields] AND "failure"[All Fields]) OR "heart failure"[All Fields]) OR "kidney"[MeSH Terms] | 2,460,520 |
| 5 | ("diabetes mellitus, type 2"[MeSH Terms] AND "adult"[MeSH Terms] AND ("Sodium glucose transport protein 2 inhibitors"[Title/Abstract] OR "sglt2 inhibitor"[Title/Abstract] OR "dapagliflozin"[Title/Abstract] OR "empagliflozin"[Title/Abstract] OR "luseogliflozin"[Title/Abstract])) NOT ("glp"[Title/Abstract] OR "liraglutide"[Title/Abstract] OR "semaglutide"[Title/Abstract] OR "exenatide"[Title/Abstract] OR "albiglutide"[Title/Abstract] OR "dulaglutide"[Title/Abstract]) | 806 |
| 4 | "glp"[Title/Abstract] OR "liraglutide"[Title/Abstract] OR "semaglutide"[Title/Abstract] OR "exenatide"[Title/Abstract] OR "albiglutide"[Title/Abstract] OR "dulaglutide"[Title/Abstract] | 18,700 |
| 3 | "Sodium glucose transport protein 2 inhibitors"[Title/Abstract] OR "sglt2 inhibitor"[Title/Abstract] OR "dapagliflozin"[Title/Abstract] OR "empagliflozin"[Title/Abstract] OR "luseogliflozin"[Title/Abstract] | 4,122 |
| 2 | "adult"[MeSH Terms] | 7,698,766 |
| 1 | "diabetes mellitus, type 2"[MeSH Terms] | 151,116 |

- 1. **Database searched: Cochrane Library [Date searched: 12/1/2022]**

| **Search number** | **Search Details** | **Results** |
| --- | --- | --- |
| #1 | (adult):ti,ab,kw | 757,097 |
| #2 | (diabetes mellitus type 2):ti,ab,kw | 50,720 |
| #3 | #1 AND #2 | 23,354 |
| #4 | (SGLT2 inhibitors):ti,ab,kw | 948 |
| #5 | (sodium glucose transport protein 2 inhibitors):ti,ab,kw | 30 |
| #6 | (empagliflozin):ti,ab,kw | 1,755 |
| #7 | (dapagliflozin):ti,ab,kw | 1,874 |
| #8 | (luseogliflozin):ti,ab,kw | 101 |
| #9 | #4 OR #5 OR #6 OR #7 OR #8 | 3,999 |
| #10 | #3 AND #9 | 1,447 |
| #11 | (GLP):ti,ab,kw | 4,656 |
| #12 | (liraglutide):ti,ab,kw | 2,295 |
| #13 | (semaglutide):ti,ab,kw | 1,000 |
| #14 | (exenatide):ti,ab,kw | 1,322 |
| #15 | (albiglutide):ti,ab,kw | 138 |
| #16 | (dulaglutide):ti,ab,kw | 532 |
| #17 | #11 OR #12 OR #13 OR #14 OR #15 OR #16 | 7,828 |
| #18 | (cardiovascular):ti,ab,kw | 88,388 |
| #19 | (heart failure):ti,ab,kw | 44,194 |
| #20 | (kidney):ti,ab,kw | 64,193 |
| #21 | #18 OR #19 OR #20 | 171,749 |
| #22 | #17 OR #21 | 178,073 |
| #23 | #10 NOT #22 | 498 |
| #24 | #23 AND ((2012:2022[FILTER]) AND (English[Filter])) | 466 |

1. **CARDIOVASCULAR RISK REDUCTION**
   1. **Database searched: MEDLINE (PubMed) [Date searched: 12/1/2022]**

| **Search number** | **Search Details** | **Results** |
| --- | --- | --- |
| 8 | (("diabetes mellitus, type 2"[MeSH Terms] AND "adult"[MeSH Terms] AND ("Sodium glucose transport protein 2 inhibitors"[Title/Abstract] OR "sglt2 inhibitor"[Title/Abstract] OR "dapagliflozin"[Title/Abstract] OR "empagliflozin"[Title/Abstract]) AND ("cardiovascular system"[MeSH Terms] OR ("cardiovascular"[All Fields] AND "system"[All Fields]) OR "cardiovascular system"[All Fields] OR "cardiovascular"[All Fields] OR "cardiovasculars"[All Fields] OR "heart failure"[MeSH Terms])) NOT ("glp"[Title/Abstract] OR "liraglutide"[Title/Abstract] OR "semaglutide"[Title/Abstract] OR "exenatide"[Title/Abstract] OR "albiglutide"[Title/Abstract] OR "dulaglutide"[Title/Abstract])) AND ((y_10[Filter]) AND (meta-analysis[Filter] OR randomizedcontrolledtrial[Filter]) AND (humans[Filter]) AND (english[Filter])) | 159 |
| 7 | ("diabetes mellitus, type 2"[MeSH Terms] AND "adult"[MeSH Terms] AND ("Sodium glucose transport protein 2 inhibitors"[Title/Abstract] OR "sglt2 inhibitor"[Title/Abstract] OR "dapagliflozin"[Title/Abstract] OR "empagliflozin"[Title/Abstract]) AND ("cardiovascular system"[MeSH Terms] OR ("cardiovascular"[All Fields] AND "system"[All Fields]) OR "cardiovascular system"[All Fields] OR "cardiovascular"[All Fields] OR "cardiovasculars"[All Fields] OR "heart failure"[MeSH Terms])) NOT ("glp"[Title/Abstract] OR "liraglutide"[Title/Abstract] OR "semaglutide"[Title/Abstract] OR "exenatide"[Title/Abstract] OR "albiglutide"[Title/Abstract] OR "dulaglutide"[Title/Abstract]) | 325 |
| 6 | "glp"[Title/Abstract] OR "liraglutide"[Title/Abstract] OR "semaglutide"[Title/Abstract] OR "exenatide"[Title/Abstract] OR "albiglutide"[Title/Abstract] OR "dulaglutide"[Title/Abstract] | 18,700 |
| 5 | "diabetes mellitus, type 2"[MeSH Terms] AND "adult"[MeSH Terms] AND ("Sodium glucose transport protein 2 inhibitors"[Title/Abstract] OR "sglt2 inhibitor"[Title/Abstract] OR "dapagliflozin"[Title/Abstract] OR "empagliflozin"[Title/Abstract]) AND ("cardiovascular system"[MeSH Terms] OR ("cardiovascular"[All Fields] AND "system"[All Fields]) OR "cardiovascular system"[All Fields] OR "cardiovascular"[All Fields] OR "cardiovasculars"[All Fields] OR "heart failure"[MeSH Terms]) | 356 |
| 4 | "Sodium glucose transport protein 2 inhibitors"[Title/Abstract] OR "sglt2 inhibitor"[Title/Abstract] OR "dapagliflozin"[Title/Abstract] OR "empagliflozin"[Title/Abstract] | 4,105 |
| 3 | "diabetes mellitus, type 2"[MeSH Terms] | 151,116 |
| 2 | "cardiovascular system"[MeSH Terms] OR ("cardiovascular"[All Fields] AND "system"[All Fields]) OR "cardiovascular system"[All Fields] OR "cardiovascular"[All Fields] OR "cardiovasculars"[All Fields] OR "heart failure"[MeSH Terms] | 2,074,201 |
| 1 | "adult"[MeSH Terms] | 7,698,766 |

- 1. **Database searched: Cochrane Library [Date searched: 12/1/2022]**

| **Search number** | **Search Details** | **Results** |
| --- | --- | --- |
| #1 | (adult):ti,ab,kw | 757,097 |
| #2 | (diabetes mellitus type 2):ti,ab,kw | 50,720 |
| #3 | #1 AND #2 | 23,354 |
| #4 | (SGLT2 inhibitors):ti,ab,kw | 948 |
| #5 | (sodium glucose transport protein 2 inhibitors):ti,ab,kw | 30 |
| #6 | (empagliflozin):ti,ab,kw | 1,755 |
| #7 | (dapagliflozin):ti,ab,kw | 1,874 |
| #8 | #4 OR #5 OR #6 OR #7 | 3,932 |
| #9 | (GLP):ti,ab,kw | 4,656 |
| #10 | (liraglutide):ti,ab,kw | 2,295 |
| #11 | (semaglutide):ti,ab,kw | 1,000 |
| #12 | (exenatide):ti,ab,kw | 1,322 |
| #13 | (albiglutide):ti,ab,kw | 138 |
| #14 | (dulaglutide):ti,ab,kw | 532 |
| #15 | #9 OR #10 OR #11 OR #12 OR #13 OR #14 | 7,828 |
| #16 | (cardiovascular):ti,ab,kw | 88,388 |
| #17 | (heart failure):ti,ab,kw | 44,194 |
| #18 | #16 OR #17 | 120,802 |
| #19 | #3 AND #8 AND #18 | 720 |
| #20 | #19 NOT #15 | 677 |
| #21 | #23 AND ((2012:2022[FILTER]) AND (English[Filter])) | 533 |
