# Supplementary material for: Expanding access to sodium-glucose cotransporter 2 inhibitors (SGLT2i) in the Ministry of Health Malaysia – a multiple HTA approach
Source: Int J Technol Assess Health Care. 2024 Dec 5;40(1):e69. doi: 10.1017/S0266462324000643 (PMC11703615; doi:10.1017/S0266462324000643)
Supplement: Choo et al. supplementary material 2 — Choo et al. supplementary material [file S0266462324000643sup002.docx]

**Supplementary Materials 2**

**Inclusion criteria**

1. Glycemic control

- Comparative studies on efficacy and safety between the three SGLT2i (empagliflozin, dapagliflozin and luseogliflozin) when given as a combination therapy with other glucose lowering drugs in adult patients with T2DM*.
- Efficacy outcomes of interest were reduction in HbA1c, fasting plasma glucose level, proportion of patients achieving HbA1c of less than 7% and reduction in body weight while safety outcomes were risk of hypoglycemia, urinary tract infections, genital tract infections, euglycemic ketoacidosis, amputations and bone fracture.

1. Cardiovascular risk reduction

- Studies comparing the efficacy of empagliflozin and dapagliflozin with each other and/or standard of care therapy in adult patients with T2DM*.
- Outcomes were reduction in risk of major adverse cardiovascular events, cardiovascular death, all-cause mortality and hospitalization due to heart failure.

1. Study design: Randomized controlled trial, meta-analysis, network meta-analysis

*Non-comparative studies were included if comparative evidence was not available.

**Exclusion criteria**

Observational studies, conference abstracts and non-human studies were excluded.
