# Supplementary material for: Expanding access to sodium-glucose cotransporter 2 inhibitors (SGLT2i) in the Ministry of Health Malaysia – a multiple HTA approach
Source: Int J Technol Assess Health Care. 2024 Dec 5;40(1):e69. doi: 10.1017/S0266462324000643 (PMC11703615; doi:10.1017/S0266462324000643)
Supplement: Choo et al. supplementary material 3 — Choo et al. supplementary material [file S0266462324000643sup003.docx]

**Supplementary Materials 3**

Records excluded
(n = 535)

Records screened
(n = 592)

Records after duplicates removed
(n = 592)

## Identification

## Eligibility

## Included

## Screening

Records identified through database searching

- Pubmed (n = 159)
- Cochrane (n = 533)

Additional records identified through other sources

- Website (n = 8)
- Applicant (n = 11)

Full-text articles assessed for eligibility
(n = 57)

Full-text articles excluded, with reasons (n=39)

Irrelevant population (n=6)

Irrelevant intervention (n=1)

Irrelevant comparator (n=3)

Irrelevant outcome (n=18)

Inappropriate study design (n=6)

Duplicate (n=2)

Study superseded by updated NMA/study (n=3)

**Figure S1.** PRISMA flow diagram of study selection for cardiovascular risk reduction

Studies included in qualitative synthesis
(n = 18)

Abbreviation: NMA – network meta-analysis

**Table S1:** Summary on characteristics of included studies

| **Author (year)** | **Study design** | **Number of included studies or participants** | **Intervention^a^** | **Comparator^a^** | **Study period/Length of follow-up** | **Outcomes reported** |
| --- | --- | --- | --- | --- | --- | --- |
| **Indication (a): Glycemic control** | | | | | | |
| **Mearns et al. (2015)** | NMA | 62 RCTs (32,185 participants) | DAPA+MTF | EMPA+MTF | Length of follow-up in included RCTs: 12 to 52 weeks | HbA1c reduction  Weight reduction  Hypoglycemic risk  UTI  GTI |
| **Seino et al.**  **(2015)** | Pooled analysis of 2 randomized trials | 2 trials (708 participants; on LUSE+MTF – 117 participants) | LUSE+MTF | None | 52 weeks | HbA1c reduction  Weight reduction |
| **Shyangdan et al.**  **(2016)** | NMA | 13 RCTs (6,517 participants)*  *Reflecting trials included in NMA for SGLT2i used as dual therapy | EMPA+MTF | DAPA+MTF | Length of follow-up in included RCTs: Minimum 12 weeks | HbA1c reduction  % with HbA1c <7.0%  Weight reduction  Hypoglycemic risk  UTI  GTI |
| **Zaccardi et al. (2016)** | NMA | 38 RCTs (23,997 participants)*  *9 RCTs assessed SGLT2i added to diet plus physical activity | DAPA+/-GLD | EMPA+/-GLD | Length of follow-up in included RCTs: Minimum 24 weeks | HbA1c reduction  FPG reduction  Weight reduction  Hypoglycemic risk  UTI  GTI |
| **Tang et al. (2016)** | NMA | 38 RCTs (30,384 participants)  *6 RCTs assessed SGLT2i as monotherapy | EMPA+/-GLD | DAPA+/-GLD | Length of follow-up in included RCTs: Minimum 24 weeks | Risk of bone fractures |
| **Azharuddin et al. (2018)** | NMA | 40 RCTs (30,384 participants)  *7 RCTs assessed SGLT2i as monotherapy | EMPA+/-GLD | DAPA+/-GLD | Length of follow-up in included RCTs: Minimum 24 weeks | Risk of bone fractures |
| **Indication (b): Cardiovascular risk reduction** | | | | | | |
| **Zinman et al. (2015)**  *EMPAREG-OUTCOME trial* | RCT | 7,020 participants  (EMPA: 4,687 participants; PLC: 2,333 participants) | EMPA+SoC | PLC+SoC | Median duration of follow-up: 3.1 years | 3-point MACE  CV death  All-cause mortality  HHF |
| **Kaku et al. (2017)** | Sub-analysis of outcomes of EMPAREG OUTCOME in Asian participants | 1,517 participants  (EMPA: 1,006 participants PLC: 511 participants) | EMPA+SoC | PLC+SoC | Median duration of follow-up: 3.1 years | 3-point MACE  CV death  All-cause mortality  HHF |
| **Kosiborod et al. (2017)** | MA | 5 RCTs (320 participants)*  **Involving* *RCTs of Dapagliflozin 10mg vs placebo in T2DM participants with concomitant HF* | DAPA+SoC | PLC+SoC | Length of follow-up in included RCTs: 52 weeks | 3-point MACE  HHF |
| **Saad et al. (2017)** | MA | 81 RCTs (37,195 participants) | EMPA+SoC  DAPA+SoC | PLC +SoC | Not available | CV death  All-cause mortality |
| **Fitchett et al. (2018)** | Post-hoc analysis of participants without baseline HF in EMPAREG OUTCOME stratified by HF risks | 6,798 participants   - N (Low to average risk): 4,226 participants - N (High risk): 1,527 participants - N (Very High Risk): 319 participants | EMPA+SoC | PLC+SoC | Median duration of follow-up: 3.1 years | CV death  HHF |
| **Verma et al.**  **(2018a)** | Post-hoc analysis of participants in EMPAREG OUTCOME stratified by with/without PAD | 7,020 participants   - N (PAD): 1,461 participants - N (Without PAD): 5,559 participants | EMPA+SoC | PLC+SoC | Median duration of follow-up: 3.1 years | 3-point MACE  CV death  All-cause mortality  HHF |
| **Verma et al. (2018b)** | Post-hoc analysis of participants in EMPAREG OUTCOME stratified by with/without history of CABG | 7,020 participants   - N (CABG): 1,738 participants - N (Without CABG):   5,282 participants | EMPA+SoC | PLC+SoC | Median duration of follow-up: 3.1 years | 3-point MACE  CV death  All-cause mortality  HHF |
| **Usman et al., (2018)** | MA | 35 RCTS (34,987 participants) | EMPA+SoC  DAPA+SoC | PLC +SoC | Length of follow-up in included RCTs: Minimum 24 weeks | 3-point MACE  All-cause mortality  HHF |
| **Wanner et al., (2018)** | Sub-analysis of participants in EMPAREG OUTCOME with/without prevalent kidney disease at baseline | 6,968  participants   - N (with prevalent kidney disease): 2,250 participants - N (without prevalent kidney disease):   4,718 participants | EMPA+SoC | PLC+SoC | Median duration of follow-up: 3.1 years | CV death  All-cause mortality  HHF |
| **Fitchett et al. (2019)** | Post-hoc analysis of participants in EMPAREG OUTCOME stratified by with/without MI or stroke | 7,020 participants   - N (MI/Stroke): 4,566 participants - N (Without MI/Stroke): 2,454 participants | EMPA+SoC | PLC+SoC | Median duration of follow-up: 3.1 years | 3-point MACE  CV death  All-cause mortality  HHF |
| **Furtado et al. (2019)** | Post-hoc analysis of participants in DECLARE TIMI-58 stratified by history of MI | 17, 160 participants   - N (MI): 3,584 participants - N (without MI): 13,576 participants | DAPA+SoC | PLC+SoC | Median duration of follow-up: 4.2 years | 3-point MACE  CV death  All-cause mortality  HHF |
| **Kato et al. (2019)** | Post-hoc analysis of participants in DECLARE TIMI-58 stratified by ejection fraction (EF)   - N (HFrEF): 671 participants - N (HF without rEF): 1,316 participants - N (No HF): 15,173 participants | 17, 160 participants   - N (HFrEF): 671 participants - N (HF without rEF): 1,316 participants - N (No HF): 15,173 participants | DAPA+SoC | PLC+SoC | Median duration of follow-up: 4.2 years | CV death  All-cause mortality  HHF |
| **Wiviott et al. (2019)**  *DECLARE-TIMI 58 trial* | RCT | 17, 160 participants  (DAPA: 8,582 participants PLC: 8,578 participants) | DAPA+SoC | PLC+SoC | Median duration of follow-up: 4.2 years | 3-point MACE*  CV death*  All-cause mortality*  HHF*  *Outcomes were also reported for subgroup with established ASCVD |
| **Bonaca et al. (2020)** | Post-hoc analysis of DECLARE TIMI-58 with participants stratified by PAD status | 17, 160 participants   - N (PAD): 1,025 participants - N (without PAD): 16,135 participants | DAPA+SoC | PLC+SoC | Median duration of follow-up: 4.2 years | 3-point MACE  CV death  HHF |
| **Inzucchi et al. (2020)** | Post-hoc analysis of participants in EMPAREG OUTCOME stratified by CVD risk factor control at baseline | 6,935 participants   - N (0-3 factors controlled): 884 participants - N (4-5 factors controlled): 3,895 participants - N (6-7 factors controlled): 2,156 participants | EMPA+SoC | PLC+SoC | Median duration of follow-up: 3.1 years | 3-point MACE  CV death  HHF |
| **Pellicori et al. (2020)** | Post-hoc analysis of participants in EMPAREG OUTCOME stratified by with/without HF | 7,020 participants   - N (HF): 706 participants - N (Without HF): 6,314 participants | EMPA+SoC | PLC+SoC | Median duration of follow-up: 3.1 years*  **This post-hoc analysis focused on early benefits measured at 1 year after randomisation* | HHF |
| **Verma et al. (2020)** | Post-hoc analysis of participants in EMPAREG OUTCOME stratified by baseline HF risk based on Thrombolysis In Myocardial Infarction Risk Score for Heart Failure in Diabetes categories (TRS-HF_DM_) | 6,952 participants   - N (Low intermediate risk): 3,429 participants - N (High risk): 1,807 participants - N (Very High Risk): 1,716 participants | EMPA+SoC | PLC+SoC | Median duration of follow-up: 3.1 years* | 3-point MACE  CV death  All-cause mortality  HHF |
| **Odutayo et al. (2021)** | NMA | 53 RCTs (88,390 participants) | DAPA | EMPA | Length of follow-up in included RCTs: 24 weeks | CV death  All-cause mortality  HHF |

Abbreviations: % - percentage; 3-point MACE – 3-point major adverse cardiovascular event (Composite of death from cardiovascular causes, nonfatal MI or nonfatal stroke); ASCVD – atherosclerotic cardiovascular disease; CABG – coronary artery bypass grafting; CV – cardiovascular; CVD – cardiovascular disease; EF – ejection fraction; FDA – Food and Drug Administration; DAPA – dapagliflozin; EMPA – empagliflozin; EU – European Union; FPG – fasting plasma glucose; GLD – glucose lowering drug; GTI – genital tract infections; HbA1c – glycated hemoglobin; HF – heart failure; HFrEF – heart failure with reduced ejection fraction; HHF – hospitalization due to heart failure; LUSE – luseogliflozin; MA – meta-analysis; MI – myocardial infarction; MTF – metformin; NMA – network meta-analysis; PAD – peripheral arterial disease; PLC – placebo; RCT – randomized controlled trial; rEF – reduced ejection fraction; UTI – urinary tract infections

1. Only intervention(s) /comparator(s) relevant to the review are specifically listed here.

**Table S2:** Summary of comparative safety between SGLT2i

| **Outcomes measured** | **Study** | **OR or RR (95% CI)** | | |
| --- | --- | --- | --- | --- |
|  |  | **DAPA vs EMPA** | **EMPA vs LUSE** | **DAPA vs LUSE** |
| **Hypoglycemia** | *Mearns et al., 2015* | RR: 1.90  (0.44 to 8.29) | Uncertain due to absence of published evidence | |
|  | *Zaccardi et al., 2016* | OR: 0.83  (0.64 to 1.08)^a^ |  |  |
| **Urinary tract infections** | *Mearns et al., 2015* | RR: 1.52  (0.89 to 2.50) | Uncertain due to absence of published evidence | |
|  | *Zaccardi et al., 2016* | **OR: 1.39**  **(1.07 to 1.81)^a^** |  |  |
| **Genital tract infections** | *Mearns et al., 2015* | RR: 1.52  (0.89 to 2.50) | Uncertain due to absence of published evidence | |
|  | *Zaccardi et al., 2016* | OR: 1.31  (0.81 to 2.21)^a^ |  |  |
| **Bone fractures** | *Azharuddin et al., 2018* | OR: 1.30  (0.26 to 8.90)^b^ | Uncertain due to absence of published evidence | |
|  | *Tang et al., 2016* | OR: 1.38  (0.72 to 2.64) |  |  |

*Statistically significant results are bolded.*

Abbreviations: DAPA – dapagliflozin; EMPA – empagliflozin; LUSE – luseogliflozin; OR – odds ratio; RR – relative risks

1. For comparison between Dapagliflozin 10mg with Empagliflozin 25mg
2. Figure reported is for comparison between Empagliflozin with Dapagliflozin and the values are reported as Odds Ratio (95% Credible Interval)
